# “The rich will always be able to dispose of their waste”: a view from the frontlines of municipal failure in Makhanda, South Africa

**DOI:** 10.1007/s10668-023-03363-1

**Published:** 2023-05-09

**Authors:** Marc Kalina, Ncebakazi Makwetu, Elizabeth Tilley

**Affiliations:** 1 Department of Mechanical and Process Engineering, ETH Zürich, 8092 Zurich, Switzerland; 2 School of Engineering, University of KwaZulu-Natal, Durban, 4001 South Africa; 3 Department of Sociology, Rhodes University, Makhanda, 6140 South Africa

**Keywords:** Solid waste management, Governance, South Africa, Climate change, Covid-19, Illegal dumping, SDG 3, SDG 11

## Abstract

A significant proportion of South African municipalities, who hold the mandate for providing solid waste management (SWM) services for millions of South Africans, appear to be on the brink of collapse. On the frontlines of municipal failure, the city of Makhanda, following two decades of poor governance and mismanagement, has found itself unable to fulfil its mandate, with the state retreating on SWM service provision, and disruptions to waste management services becoming a daily reality. Drawing on embedded, qualitative fieldwork, this article examines how differently placed residents have experienced disruptions to SWM services. This work explores how residents of Makhanda’s two halves: the affluent and predominantly white neighbourhoods in the west, and the poor, non-white townships in the east, have (or have not) adapted to manage and dispose of their own waste during periods of disruption. Findings suggest that disruptions to waste management service provision have been broadly experienced by residents. However, the consequences of interruptions to municipal collection have not been evenly borne, as more resourced, western residents have been more successful at managing their own waste disposal, while the residents of Makhanda’s townships are less capable of coping, with affected communities coming to resemble a dumping ground, and residents having to adopt unsafe or environmentally harmful disposal practices. These findings are important because they shed light on the challenges of creating cleaner, more equal communities without healthy municipal participation in waste management services, while raising important considerations for a South Africa facing the possibility of widespread municipal collapse.

## Introduction

Makhanda, formerly known as Grahamstown, in South Africa’s province of the Eastern Cape, feels very much like a college town. Dominated to the west by the sprawling, and park-like campus of Rhodes University, the town and University intermingle in a plethora of heritage spaces and historic buildings. The compact and busy city centre is outfitted with all the accoutrement of a college town: art galleries, pubs, a small but prosperous professional class, a vibrant student body, and festival scene. The town itself was founded in 1812 as a far-flung fort, at what was then the furthest reaches of the British Empire in Southern Africa. Once an outpost of colonialism, the town retains its colonial air, with a High Street and Market Street straddling the historic centre, connecting prestigious private schools, graceful and manicured estates, dozens of aged heritage churches, and the historical remnants of British colonial administration, which are now some of the town’s most visited tourist attractions. Yet, ascending from the city bowl, towards the east, away from the city centre and the comfortable western neighbourhoods, with names like Sunnyside and Somerset Heights, one would be instantly reminded that colonial ‘charm’ is a loaded concept in South African cities. Makhanda’s genteel spaces have been shaped by centuries of colonialism and apartheid: by racial exclusion which forced Makhanda’s non-white residents to the city’s fringes. Like the story of most South African towns, Makhanda’s remains a tale of two cities. Makhanda was constructed to be physically divided along racial lines, with the non-white residents of Grahamstown East, in the sprawling Townships of Joza, Tantyi, Hlalani, and Fingo, markedly poorer than those in Grahamstown’s western, and predominantly white, neighbourhoods. Unfortunately, Makhanda’s historic charm does not extend to its townships, an area collectively known as Rhini,^1^ and residents suffer from a lack of economic opportunities, adequate housing, few or inconsistent basic services, and an environment far less manicured than in Makhanda’s wealthier, cleaner, and whiter, communities.

Since the start of South African democracy in 1994, one of the overriding aims of the new democratic project has been to bridge this divide and lessen the inequality that characterises South African communities. Indeed, South Africa has made major strides in addressing service delivery gaps, including extending solid waste management (SWM) services to previously underserviced communities, such as Rhini, since the end of apartheid. Much of this progress has centred in South Africa’s metropolitan municipalities,^2^ which have made impressive gains. Yet crushing inequality persists, particularly in South Africa’s small towns where apartheid area spatial orientations have largely gone unchanged. In South Africa, municipal governments are on the frontline of service provision, providing water, electricity, and waste and sanitation services to all communities. However, Makana Local Municipality, the municipality which includes Makhanda, has found itself, following two decades of poor governance and mismanagement, unable to fulfil its mandate. Sued by its own citizens for its failure to provide basic services, including solid waste collection, and placed under periods of national administration twice since 2014, the municipality is widely considered, by out-spoken residents and the national government, to be in crisis (Nowicki, 2020a, 2020b). As a result, rather than continue to extend solid waste management services into previously underserviced communities like Rhini, and improving collection in Makhanda’s western neighbourhoods, waste management services have become at best inconsistent, and at worst, non-existent, depending on what part of town you live in, and what resources Makana is able to muster on a given day.

Unfortunately, Makana’s troubles are not unique within South Africa, and the overall health of the nation’s Local Municipalities is alarming. Scholars and analysts have been ringing the alarm bells for years over a number of compounding factors within South African municipalities, such as a lack of skills, urban migration, weak supply chain management processes, poor financial management, and most glaringly, irregular spending,^3^ threatening municipal financial integrity (Kanyane, 2014). However, since 2020 the municipal crisis seems to have reached a tipping point. For the 2019/2020 financial year, only 27 of the 257 municipalities in the country, less than 11 per cent, received clean audits, while the ongoing Covid-19 crisis has put further strain on municipal resources (Mabuza, 2021). As a result, a significant proportion of South African municipalities appear to be on the brink of collapse. According to the Auditor-General (2019), 87 municipalities have been currently red-flagged as being delinquent or under administration and are at risk of collapse, while local media sources argue that even this count is too low, suggesting that another 43 should be added to that list, which, altogether, would account for almost half of South Africa’s 278 municipalities (Trench & Gerber, 2021). Despite the National Treasury signalling its own alarm about the situation and announcing a raft of measures in 2021 to assist embattled municipalities, reports submitted to Parliament^4^ admit the situation is likely to deteriorate further (Staff Writer, 2021). Makana has been the face of failure amongst South African municipalities, but what has happened there may be an indication of what is in store for millions of South Africans. As South Africa’s municipalities head towards an uncertain future, what are the implications for their service delivery mandate, and as waste management capacity deteriorates, what are the consequences for South Africa’s citizens who rely upon municipal systems daily? As the state retreats, who steps into the service delivery void, and how do citizens, including the most vulnerable, cope with disruptions?

Drawing on embedded, qualitative fieldwork within Makhanda, including 43 semi-structured interviews with residents and local stakeholders, participatory observation, and secondary analysis of local media reporting, the purpose of this research is *not* to explain or unpack the causes that have contributed to Makana’s current state. Rather, we aim to understand how differently placed residents have experienced the disruptions to Makana’s waste management services which have become a growing reality over the past decade, and accelerated over the past two years. We explore how residents of Makhanda’s two halves: the affluent and predominantly white neighbourhoods in the west, and the poor, non-white townships in the east, have (or have not) adapted to manage and dispose of their own waste during periods of disruption. We also interrogate how residents identify the externalities that have emerged from the deterioration of municipal collection, and reflect on these implications, locally and nationally, for the South African goal of creating more equal communities. This work responds to a gap in the literature on waste management service delivery, which has well-documented the hollowing out of municipal services, globally, through neoliberal formations of the city and privatisation (Mathekganye et al., 2019; Samson, 2020; Yates & Harris, 2018), what Lobao et al. (2018) refer to as the ‘shrinking state’. Authors have also accounted for municipal collapse as a result of conflict or other external pressures, and its implications for citizens (Bolton, 2020; Brinkerhoff et al., 2012; Ozanne & Ozanne, 2021), but have not adequately reckoned with the consequences of municipal incapability due to failures in governance or other internal factors, especially within a South African context where such conditions are unfortunately widespread (Kanyane, 2014). Our findings suggest that disruptions to waste management service provision in Makana have been broadly experienced by residents in Makhanda’s affluent west and its historically neglected east. However, the consequences of interruptions to municipal collection have not been evenly borne, as more resourced, western residents have been more successful at managing their own waste disposal, either through storing waste during periods of disruption, personally transporting it to the dump, or, most often, paying for private collection. In Makhanda’s townships, however, residents do not have the space to store waste, or the resources to arrange for private collection. As a result, Joza, and Makhanda’s other townships, have come to resemble a dumping ground, with municipal collection points coalescing into informal dumpsites, and residents having to adopt unsafe or environmentally harmful practices, such as illegal dumping or burning, to dispose of household waste. As noted, these findings are important because they shed light on the challenges of creating cleaner, more equal communities without healthy municipal participation in waste management services, and raise important considerations for a South Africa facing the possibility of widespread municipal collapse. Inequality already underpins access to waste management systems in South Africa, yet these findings hint at the consequences for South African urban spaces when access to waste management becomes more unequal, and the state retreats on its service delivery mandate. We highlight the role of climate change in exacerbating these inequalities, and hastening municipal collapse, while emphasising the need to support innovative, decentralised waste management solutions in vulnerable communities, which do not predicate on the participation of a robust state partner, nor give way to neoliberal privatisation.

## Situating municipal failure

The retreat, or collapse, of the South African state at the municipal level has grave implications for waste management service provision, and service delivery more broadly. Waste management literature has provided ample, grounded discussion, on the extension or upgrading of municipal service provision in the Global South (Baptista, 2019; França et al., 2020; Rai et al., 2019), including within South Africa (Bhorat et al., 2012; Kroukamp & Cloete, 2018; Ndevu & Muller, 2018; Rasmeni & Madyira, 2019). Yet, there have been few discussions about what happens when services deteriorate or break down altogether. The consequences of municipal bankruptcy have received some attention (Gillette, 2019; Peck, 2014; Turco, 2015), but with scant mention of the pain this can bring to individual citizens. More common have been conversations around the hollowing out of public service provision through encroaching neoliberalism. For instance, Lobao et al. (2018) speak to the ‘shrinking state’, i.e. state retreat orchestrated through the withdrawal of finances, staff, and ultimately services, spurred by a failure to increase resources to match growing demands, and growing private sector involvement in service provision. Neoliberal privatisation has been observed to degrade the quality of public services, especially in low-income areas (Navarrete-Hernandez & Toro, 2019), and work to the exclusion of those unable to pay, restricting access to formerly public goods and spaces (Smith, 2021). In addition, privatisation has been reported to produce negative impacts on environmental sustainability locally (Fasenfest, 2021; Homsy, 2020), but may result in improvements to public service work ethics (Maron, 2021). Moreover, although there have been calls for remunicipalisation of basic services (McDonald, 2018), Santos (2021) argues that privatisation is akin to a one-way street, and that the restatisation of services can be difficult once the private sector has been admitted.

Within South Africa, neoliberal approaches to public service provision, and water provision in particular, have been observed to act to the detriment of the poor, who cannot afford reconfigured private services, contributing to inequality in services received, especially over the long-term (Mathekganye et al., 2019; Yates & Harris, 2018). Regarding waste management services, neoliberal privatisation remains in its infancy, but has begun to make in-roads in some South African Metros—to the detriment of the most vulnerable. For instance, Storey (2020) examines neoliberal formations of the city in Cape Town as ‘discourses of blame’, framing the interactions of the poor with waste, such as dumping, as irresponsible and illegal, and implying that residents are to blame for service limitations, justifying further restrictions and reinforcing historic spatial inequality. Likewise, the commodification of waste, and the opening of recycling markets to private companies in Johannesburg, has contributed to the dispossession of informal waste workers, transferring their livelihoods to formal actors (Samson, 2011, 2020; Samson et al., 2022), a phenomenon that has also been observed globally (Dinler, 2016; Hartmann, 2018; Luthra, 2019, 2020).

Conversely, the fragility of the state, and its impacts on municipal service delivery have been explored in relation to conflict zones, with an emphasis on restoring services post-conflict (Bolton, 2020; Brinkerhoff et al., 2012), and on so-called failed or collapsed states, though the level of that analysis is generally at the national or federal level, rather than with municipalities (Call, 2011; Rotberg, 2003). Furthermore, the impacts (and predicted impacts) of climate change, and catastrophic climate change-related weather events on services and service infrastructure have also received considerable attention (Chirisa et al., 2016; Ozanne & Ozanne, 2021; Ranghieri & Ishiwatari, 2014; Shi & Varuzzo, 2020), including within South Africa (Jozipovic, 2015; Piketh et al., 2013). In addition, in South Africa, conversations around the degradation of public services have been bound in discussion of civil unrest, i.e. on service delivery protests, the role of poor services in driving protest, as well as the impact of protest action on disrupting service provision (Khambule et al., 2019; Netswera, 2014). However, the consequences of more recent, widespread and disruptive episodes of strife, such as the wave of civil unrest that washed over the country’s cities in July 2021, accounting for the most severe violence that South Africa has experienced since the end of apartheid, have yet to be fully unpacked, despite initial commentaries (Kalina, 2020, 2021). Regardless of this valuable scholarship, we still lack a useful frame of reference for the type of local state-failure that is taking shape in Makhanda and elsewhere. Perspectives on Cape Town’s water crisis in the mid to late-2010s give some insight into the consequences of when services, quite literally, dry up (Haque et al., 2021; Millington & Scheba, 2021), but do not compare to the possible impacts of a broad base collapse of municipal services, and do not speak to waste management services at all (although water crises can profoundly affect SWM systems, and vice versa). Yet, regardless of the source of state ‘failure’, be it through neoliberalism, disaster, climate change, or civil unrest, we lack grounded accounts of how these failures reverberate through communities: how a failure to provide services impacts citizens, especially the most vulnerable. Although there is mounting evidence that the retreat of the state will drive inequality, particularly in a fragile South Africa, we lack a clear understanding of how these inequalities manifest, and become entrenched, across the landscape of its divided small towns.

## Methodology

**Fig. 1:**
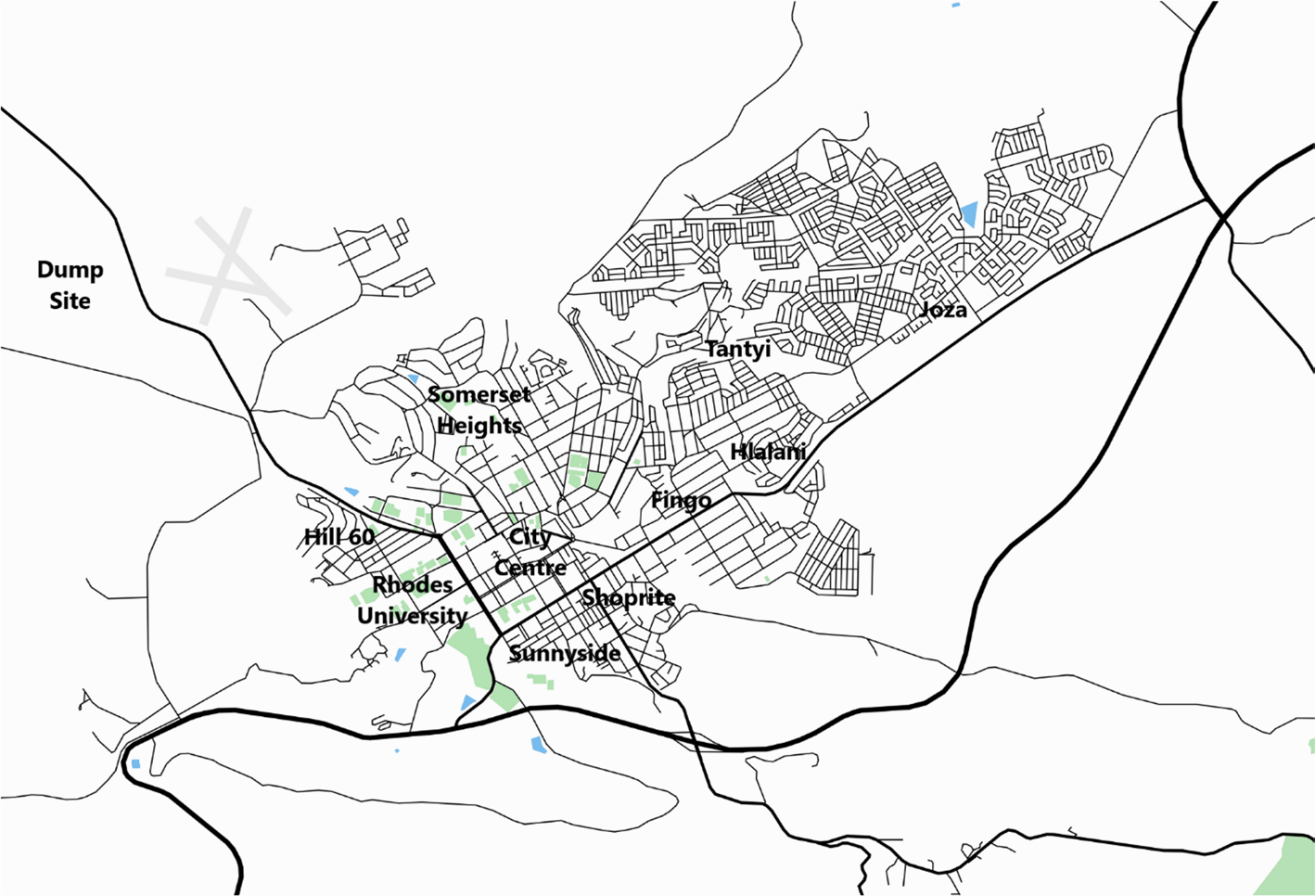
Makhanda (Map data source: Mappin WMS)

The overriding purpose of this investigation was to understand how differently placed Makhanda citizens experience and cope with disruptions to municipal solid waste collection. To meet this aim, 43 semi-structured interviews were conducted with Makhanda residents and key stakeholders from the Makana Municipality and civil society between June and October 2021. These interviews have been supplemented by substantial participatory observation with Makhanda’s communities, and secondary analysis of local media reporting. As described, Makhanda is physically divided along socioeconomic and racial lines: between relatively wealthy and historically white west, and the poor, overwhelmingly non-white townships to the east. Twenty interviews were conducted with residents of western Makhanda, in the affluent neighbourhoods of Hill 60, Somerset Heights, and Sunnyside. Twenty more interviews were conducted in eastern Makhanda, centring on the township of Joza (Fig. 1). Prior to this principal data collection, interviews were conducted with a managerial-level official from Makana overseeing solid waste collection, who provided gatekeeper approval necessary for ethical clearance, with representatives of the Makhanda Residents Association (MRA) and with leadership from the Unemployed People’s Movement (UPM) who were important gatekeepers to residents of Joza.

All communities and respondents were purposively selected to meet the criteria of the study, i.e., to obtain a broad sample of Makhanda’s socio-economic and racial groups. Within eastern Makhanda, Joza was specifically selected from Makhanda’s four townships, as it was where the researchers had previously established research connections and could therefore easily negotiate access through the assistance of the UPM. Participants were solicited through a door-to-door research methodology, where households were purposively selected based on their availability and willingness to provide consent. Residents were interviewed on their doorstep, face-to-face, and with only interview occurring per household. Homes were purposively selected within each community, with an effort made in Makhanda’s western neighbourhoods to interview a roughly equal number of residents from each of the three areas visited (Hill 60, Somerset Heights, and Sunnyside). The decision to conduct interviews outdoors, in front of homes, was further motivated by safety protocols necessitated by the on-going Covid-19 pandemic.

Interviews were semi-structured, with questions centred on wasting and waste management behaviour, experiences with service delivery disruptions, and coping strategies. Interviews averaged roughly 25 min in duration. As noted, 40 interviews were conducted, across Makhanda’s eastern and western halves, which was the maximum feasible number that could be conducted with the time and resources available; however, the consistency of respondents’ responses suggests that saturation was achieved. Interviews were conducted, by the second author, in the respondents preferred local language (isiXhosa in Joza, and predominantly English in Hill 60, Somerset Heights, and Sunnyside). Interviews were audio recorded, and later transcribed into English. Participation was voluntary, and written, informed consent was gained from each respondent before every interview. Responses were recorded anonymously. The study has ethical approval by the University of KwaZulu-Natal (UKZN) Humanities and Social Sciences Research Ethics Committee, Protocol No. HSSREC/00002671/2021. Data were analysed thematically and stored, transcribed, and then coded within the software programme Nvivo.

## Findings

### Experiencing disruption

How have residents experienced Makana’s troubles, and the resultant disruptions to SWM services? As it stands at the time of the interviews, and as we will hear from residents, municipal waste collection across Makhanda broadly has become at best inconsistent, and at worst, non-existent, depending on what part of town you live in, and what resources the city is able to muster on a given day. Although Makana’s troubles have compounded in recent years, services have not deteriorated overnight. Historically, the city centre and surrounding western neighbourhoods enjoyed regular, weekly, waste collection, and in the decade after the start of democracy in 1994, steady gains were made in extending and systemising collection in the townships to the east. So how did we get to this point? As early as 2002, the Makana Municipal Diagnostic Study on Infrastructure Investment and Finance noted a deterioration in refuse services, reporting that the quality of service in former Black areas seemed to be particularly poor and there was a lack of acceptable standards in the town centre (Makana Local Municipality, 2002). Furthermore, a scan of local news media highlights a large uptick in illegal dumping (or at least reporting on illegal dumping), particularly in the city centre, with residents describing a proliferation of new dumping sites, and the cities failure to clear them,^5^ a trend which Etengeneng (2012) attributes to a lack of black refuse bags (which Makana had previously provided) and the insufficiency of disposal sites in the townships. Between 2009 and 2011, the first reported^6^ strike actions cause significant disruption to services. In 2009, municipal waste workers, striking over wages, covered the roads in litter, dumped rubbish in front of businesses, and stopped work. In the aftermath, citizens and business owners had to clean up and take the rubbish to the municipal dumping site in their own vehicles. In 2010 there was another municipal strike, with workers emptying bins around town including dumping trash outside City Hall, and in 2011 there was another strike by municipal waste workers that, once again, left the streets of Makhanda covered in rubbish.

Looking at the historical narrative, this period of strike action seems to mark an acceleration in the decline of public services, with reporting on infrequent waste collection becoming more regular, even in Makhanda’s wealthier neighbourhoods, after 2009. For instance, in 2010 Grocott’s Mail^7^ reported on the formation of an illegal dumping site in Somerset Heights (Zimkhitha, 2010). Predictably, residents were alarmed, calling on the municipality for weeks to have it cleared, and threatening to hire a private contractor to clear the site while sending the bill to Makana. The municipality eventually had it cleared after a month, however the growing inequalities which have characterised Makana’s breakdown came to the surface: by this point illegal dumpsites had become common and permanent, in the townships, with reporting, like an article in Grocott’s Mail (2013), which described people who live near municipal rubbish collection points in the townships as suffering because collection points were turning into permanent dumps from irregular collection. Yet, the appearance of one in a predominantly white neighbourhood was a cause for alarm, with residents willing to resort to a private contractor, but eventually succeeding in pressuring the municipality to clear it.

In 2014 Makana was put under its first period of administration^8^ by then Minister of Cooperative Governance & Traditional Affairs (Cogta), Pravin Gordhan, for a period of 3 months, to halt what was seen by the national government, as an alarming decline in financial and infrastructural health. Shortly after being placed under administration, a highly critical forensic report, known as the Kabuso Report (2015), publicly named several Makana officials purported to be involved in financial impropriety. The period of provincial administration did little to turn around Makana’s troubles, however. In 2014, municipal workers held a 2 weeks strike which stopped services, prompting a resident to take their waste and dump it outside the Mayor’s office (which is a familiar protest tactic in Makhanda, see Kalina, 2021). Also in 2014, residents observed illegal dumpsites continuing to proliferate, with 208 sites being reported in the eastern sections of the town and in the townships (Ball et al., 2014). And by 2017 the Democratic Alliance (DA)^9^ warned of Makana's growing inability to manage waste in the city, reporting that illegal dumpsites have appeared all over the municipality, including near schools, next to homes, near sports fields (Carlisle, 2017).

2018 saw an acceleration of civic unrest directed towards the municipality, and unprecedented breakdowns in municipal governance. In late 2018 rallies by local civil society organisations succeeded in forcing the then current mayor’s resignation, and intense protest action (Kalina, 2021), ahead of the visit of President Cyril Ramaphosa, were intended to send a strong message of dissatisfaction to the national government. In 2019 the Unemployed People's Movement (UPM) and other civil society organisations brought an application to the high court to have the Municipality dissolved, and in 2020 the High Court in Makhanda on Tuesday ordered that the Makana Municipality be dissolved and placed under administration for violating its constitutional mandate by failing to provide basic services to the community, and dissolving the municipal council until fresh elections in 2021, which was a first in South Africa, with the High Court ruling that the municipalities failure to provide services to the residents of Makhanda was unconstitutional (Ellis, 2020). Nonetheless, in late 2020 the residents of Makhanda took Makana Municipality to court because of neglect and inconsistent waste management. In court, Makana placed the blame on the residents and made arguments to exonerate themselves, but in 2021 Makana was found to be in breach of the court and was ordered to clear illegal dumpsites.^10^ The municipality was also ordered to resume providing rubbish bags to households weekly, and produce a comprehensive audit of Makhanda's waste management needs, and address findings within 6 months (High Court of South Africa, 2021). Yet, later in 2021, although Makana claimed to have abided by the court order, residents of Makhanda’s townships were reporting that their waste remained uncollected for over year, and that municipality had once again, stopped giving out black plastic bags, while the illegal dumping sites remained in use (GroundUp, 2021).

During the 2021 local elections, a new political party, the Makana Citizens Front (MCF), a broad coalition, drawing support from residents from all parts of Makana, contested the local government elections, channelling residents’ anger at service disruptions, and although the dominant political party, the African National Congress (ANC), maintained control of the municipality, the MCF won 5 seats on council, becoming the official opposition in the municipality. This new balance of power has not yet resulted in concrete improvements to governance. A final constraint, and bringing our timeline up to the point of this investigation, was the ongoing Covid-19 pandemic. Nationally mandated lockdowns disrupted already irregular collection, while illness amongst municipal workers led to the municipality to frequently suspend collection for a lack of staff. Furthermore, a complete shutdown of the city in June 2021, organised by the UPM, led to the suspension of all municipal services.

Interviews with residents support the textual narrative of a gradual decline in services quality over the past decade. In the western neighbourhoods, residents described, what had once been considered reliable collection, deteriorate to what can at best be described as an inconsistent offering. One resident^11^ described how this dynamic has shifted: “we would put [our bags] on the street pavement, and they would collect it weekly. Now they come now and again. I don’t know their procedure. They come when they feel like it, I don’t even see other people putting their trash out anymore.” Another resident^12^ expressed their frustration with these inconsistencies, and in line with a broader theme, with the municipalities failure to communicate them, “[Now] they just don't pitch [show up] and there's no SMS or WhatsApp saying, you know, we're not coming…. It is very up and down, so we just deal with it as it comes.” In Joza, residents stressed that services within their community had always been poor, but had, in recent years, become even more unreliable. One resident^13^ explained, “this has been happening over many, many years…over a long period of time. Sometimes they are good…. diligent and coming weekly to collect the trash, then other times they just don’t do it.”

### Problematising failure

Expressing what often sounded like exasperation combined with resignation, respondents overwhelmingly blamed the municipality for the deterioration in services. When respondents spoke of who was to blame for the current situation, the overwhelming majority pointed to Makana as the problem, characterising the municipality as both corrupt and incompetent. Municipal leadership and publicly elected officials, such as local ward councillors in particular, came under fire from residents, primarily for their inaction towards improving services and for poor communication around disruptions, as one resident^14^ articulated:

Makana municipality is responsible, they are just not doing anything… you take on the job as councillor, or mayor, or director for the betterment of your town, they are the ones that should be in control of everything and seeing things get done and it’s not happening.

However, rarely were municipal waste workers themselves targets of blame, with many residents often sympathising with their strikes, even though it disrupted collection. A resident of Joza^15^ commiserated with workers doing their best within a collapsing municipal structure, “people are not getting paid their salaries. Money was being taken from their pensions, so they had their own grievances that are internal that they were dealing with. Obviously then impacted the service that they deliver to us.”

In addition, within both halves of Makhanda, there was strong consensus that although services had been poor in previous years, there was marked decline in both the frequency and reliability of waste collections during the still ongoing Covid-19 pandemic, beginning in South Africa in early 2020. These disruptions were largely attributed to nationally imposed lockdowns which paused collections, illness among waste collection workers which made it difficult for Makana to sufficiently staff collection routes, and protest action by municipal workers, striking over a lack of personal protection equipment (PPE) in the workplace. The following quotes illustrate the impact that Covid-19 had on collections:

Well, at the height last year 2020 that was Covid year….I think that was one of the most terrible years of service delivery in terms of waste management, waste picking up…There [was] a delay at the beginning of Covid because [of the] lockdown of essential services and also the fact that Makana workers felt like they still do not have enough protective gear to go through peoples tissues and all that kind of stuff.^16^

Before I come to work Monday, I put [the bags] outside. If I’m talking about how frequent, I would say maybe 70% of the time they do collect. But during Covid we did have challenges.^17^

When one person had Covid they would close down the whole division so that's recent. I mean that's the last two years…but it's always when they are either on strike or now recently because of the vehicles not running properly or they don't got enough vehicles.^18^

If there is Covid the whole department closes, and you sit the whole 14 days without your trash being collected. It becomes a problem because it accumulates. And with protest actions they damage service vehicles, [so] obviously they can’t come and collect.^19^

Although many residents expressed an understanding that some Covid-related disruption in weekly services was inevitable, given the scale of the pandemic and its national impact, anger was once again largely focused on the municipality for not communicating Covid-related disruptions, managing staffing requirements for what most deemed an essential service (waste collection), and for failing to protect the health and safety of municipal waste workers. One resident of Somerset Heights^20^ clearly expressed this sentiment:

I don’t think the workers are fundamentally responsible… I know it’s easy to blame and say there were protests and they were striking for safety, but if I was a rubbish collector, I would absolutely need protective gear to pick up rubbish, I would need gloves and all that stuff. So, I don’t think that is the root cause of these types of problems, I think again it’s symptoms of a corrupt administration, a broke down, morally bankrupt municipality that has gotten away with doing such things across the board. It’s very difficult to find one thing that this municipality is good at.

### Coping with disruptions

Although residents across the two halves of Makhanda’s socio-economic divide have, to different degrees, experienced disruptions due to Makana’s growing incapability, accentuated by the Covid-19 pandemic, the two communities’ abilities to cope with disruptions to collection vary dramatically, with residents of the cities wealthier, western neighbourhoods able to ‘make a plan’, and store waste until collection resumes, pay for private waste collection, or take waste to the dump themselves. However, in Joza, residents are much more financially and spatially constrained, restricting their ability to adopt the same disposal techniques as middle-class residents in the west. Rather, Joza residents have been forced to resort to less healthy disposal practices, such dumping at collection points or in the veldt,^21^ or by burning their household waste. Across Makhanda’s disparate communities, however, coping has been facilitated by the emergence or reinforcement of pre-existing community groups, active on social media, which inform members of disruptions, supplementing traditional, dysfunctional avenues of municipal communication.

In the western neighbourhoods, disruption is more of an inconvenience than a crisis, as residents have largely adapted to the inconsistencies of municipal collection and developed coping strategies that allow them to still dispose of their waste sanitarily, despite the additional time, costs, etc. which are borne by these residents. Because municipal collection still does occur within the researched western neighbourhoods, although it has grown increasingly sporadic and inconsistent, the most common coping method employed by residents was to store their waste until it can be collected. As respondents described, they still habitually put out their bags to the kerbside on trash day, but bring them back in the evening if they are not collected. Thus, even if the municipality does not show up on a given day, residents will try again for collection next week, as one respondent^22^ described, “I just keep it in my yard until the next time someone will collect… I just keep it for whenever they are coming again.” This behaviour is facilitated by the fact that western residents, largely live-in free-standing, single-family homes, and have sufficient space (yard, garage, etc.) on their property to securely store waste.^23^ One resident^24^ described how the size of their property insulated them from the impacts of disruptions, “I’ve got sufficient black bins to, uh tide me over for or for a second week or even a third week or fourth week if need be.”

When the municipality missed enough collections, and western households were no longer able to store the volume of waste they had accumulated, residents, as several described, ‘make a plan’ to dispose of it themselves, either through a trip to the dump, or by paying for private collection. Makhanda’s dump is located about three kilometres from Somerset Heights and Hill 60, heading away from town on the Craddock Road (Fig. 1). For many residents in these neighbourhoods, who predominantly own cars, a quick trip to the ‘tipping point’, was an easy solution for clearing out their waste backlog. Although these residents described disliking going to the dump, even characterising it as unsafe, it remained an accessible option during long drought periods of municipal collection. More common, however, were residents paying for private collection, either individually or with their neighbourhoods. Within western Makhanda, popular collection services have emerged, with one individual’s name in particular, becoming ubiquitous with private collection, as the following quotes show:

I’ll just simply hire someone like Neil Coetzee^25^ who drives around with a truck and collects people’s rubbish from the residential areas.^26^

You get hold of Neil he takes it away and it's not a problem.^27^

You know if your garbage isn’t collected then you pay Neil Coetzee to come and collect.^28^

My neighbours, I’m not sure if they go to the tip, but some of them make use of Neil Coetzee.^29^

Although for some, the cost of regularly utilising private collection was a barrier, most found these services affordable,^30^ and a worthwhile convenience, especially in periods of extended disruption. As a result, a large minority of respondents described no longer bothering to place their waste out for the municipality on collection days, and instead were fully relying on private services.

**Fig. 2:**
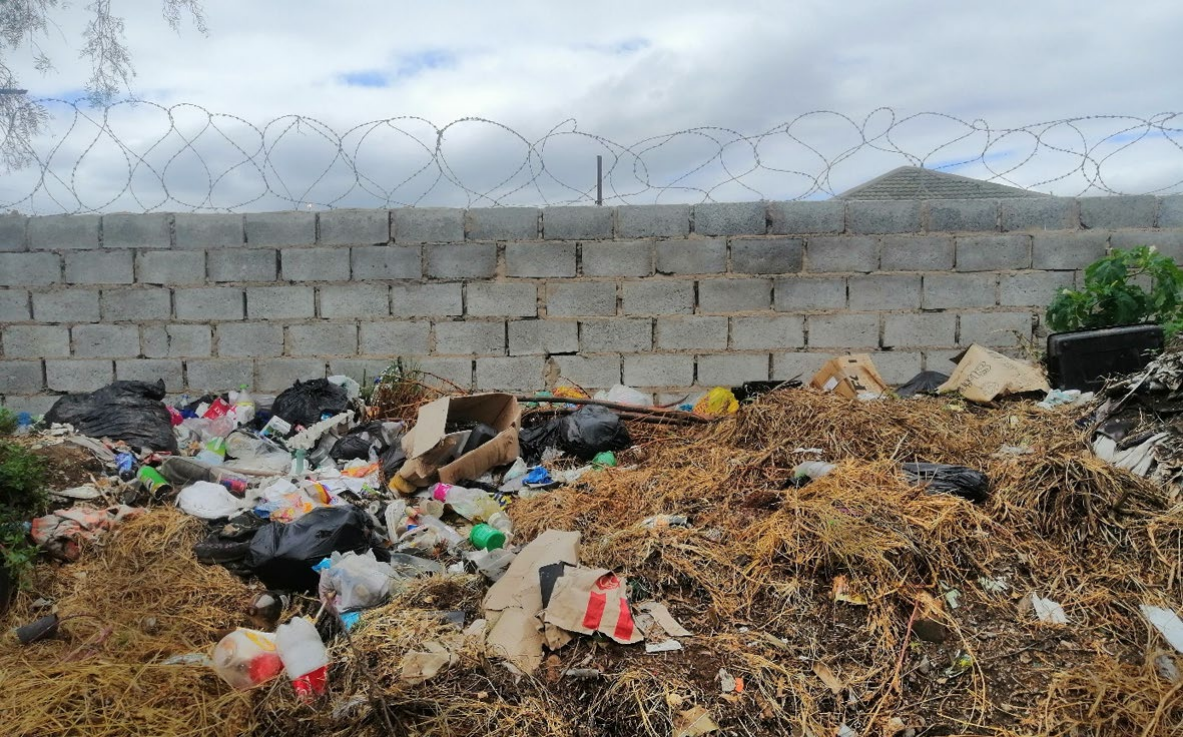
Waste accumulating along a wall in Joza (Authors)

Unfortunately, these coping strategies are out of reach for most Joza residents. In the crowded township, most lack sufficient or secure space to store their waste during collection droughts. While few own a car to transport waste themselves to the dump, the cost of paying for private collection is prohibitive.^31^ As a result, respondents described adopting less healthy disposal practices such as dumping, at collection points or in the veldt, or by burning their household waste. Illegal dumping sites are a common facet of life in South Africa’s townships, where SWM services are universally insufficient. In Joza, where collection has all-but come to a halt, dumps, however, have taken over the community, with many residents, in the west and the east, describing the townships as one large dump site. Specifically, illegal dumpsites have grown at former/current municipal collection sites, where residents continue bringing their waste in the hope that it will be collected eventually, and all across the unoccupied or marginal land that surrounds the community, such as the aforementioned veldt, in water courses, and along property lines where it accumulates against walls and fences (Fig. 2). For a large portion of the community, dumping in these spaces is the only disposal option.

**Fig. 3:**
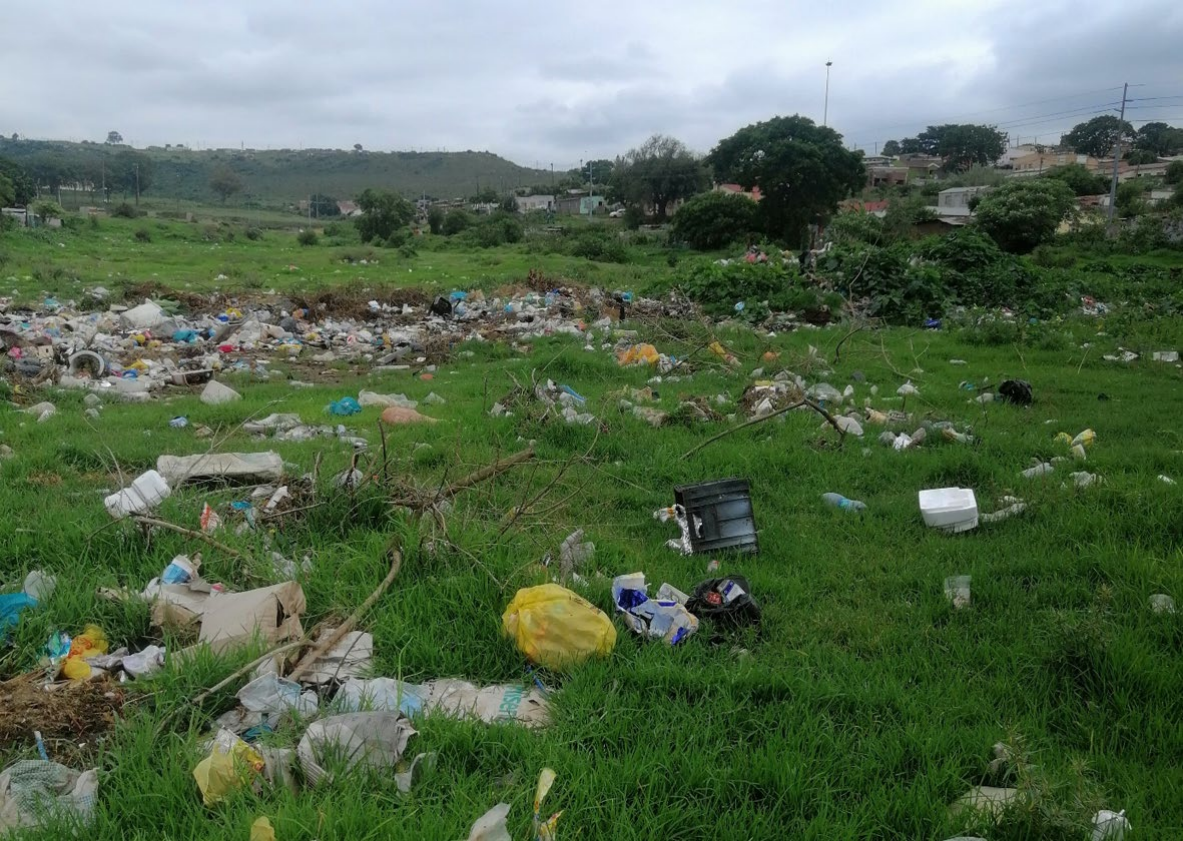
Waste in the veldt near Joza (Authors)

More than half of Joza respondents described burning their household waste, at least some of the time or all the time. However, this option poses its own risks, and is more difficult for those who live in denser parts of the community. For instance, although those who burned their waste felt it was preferable to dumping (and many expressed a desire that their neighbours who did not burn would do so, rather than dump), they described burning their waste as time-consuming and unsafe- both because of the risk of starting a larger, uncontrolled fire, and from feeling unsafe (due to crime) walking out alone into the veldt to burn (Fig. 3). One respondent,^32^ who lives in a dense part of Joza, described the constant poor choices residents are forced to make to dispose of their waste:

We can't burn it; there are no safe spaces to burn it. We also can’t go and put it in those bins. They are supposed to have bins you fill with trash that are placed in specific areas, so when they don’t come you go and place it in them. We don’t have those [here]. You see? And in the few places they do have them it’s filthy. They fill up with rubbish and no one comes to collect the trash. Once they fill up, people dump around them.

When asked how they were coping with disruptions, many Joza respondents stated bluntly that they were not coping at all, but instead surviving; making the best of an unbearable situation. Even those who managed to burn, or otherwise get their waste away from their homes, knew residents were fighting a losing battle and lamented the overgrowing piles of waste that are slowly consuming the community. Joza residents are drowning in their own waste and have no idea when they will catch a breath of air.

Across Makhanda, local, usually neighbourhood-based, community groups, active on social media, have stepped in to share information on waste collections and disruptions. In western Makhanda this has largely occurred in pre-existing neighbourhood watch groups, based on Whatsapp. Through these groups, neighbours share information on possible disruptions (such as planned protests or strikes) and inform each other when collection has not occurred on trash days, so that residents can bring their waste back inside. In Joza, Facebook groups were more popular amongst residents, but effectively functioned the same, as a platform to share information on collection. One universal complaint amongst all residents was poor communication by the municipality around disruptions. Makana has a Facebook page and does use the platform to communicate service-related issues; however, respondents described Makana’s posts on Facebook as inadequate, and a one-way channel of communication, with residents unable to effectively communicate back to the municipality about services. Finally, one coping method which does not seem to have gain much traction in either half of Makhanda is a conscious reduction by residents in production of household waste, with few respondents describing changes in purchasing behaviour or reductions in consumption since the start of waste collection disruptions.

### Impacts

The deterioration of Makana’s public waste management services have impacted all Makhanda residents. However, as the coping strategies adopted by Makhanda’s disparate communities suggests, these impacts have not had equal weight, or been equally borne. Broadly, the breakdown in waste collection has contributed to a dramatic deterioration in the cleanliness of the local environment, which is visible in all parts of Makhanda, but is most marked in the townships which have increasingly become normalised as a dumping ground.

**Fig. 4:**
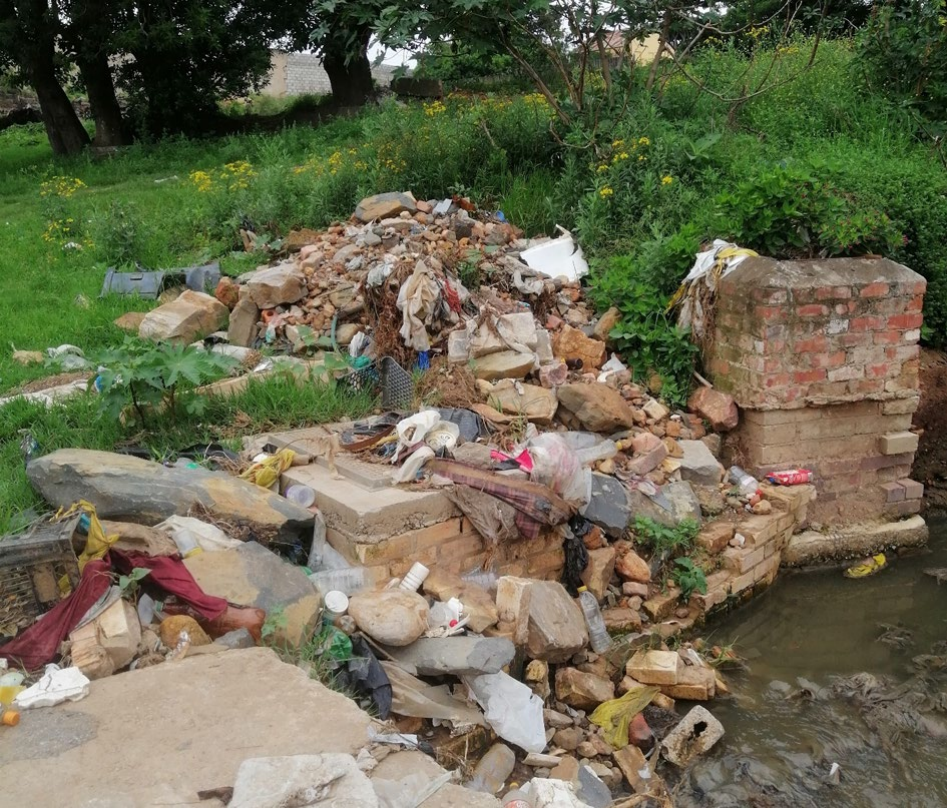
Illegal dumping near a watercourse (Authors)

In western Makhanda, although residents have largely been able to cope with disruptions at a household level, green space has become, in recent years, choked with litter. According to respondents this is due to the municipality’ inability to clear out illegal dumping sites promptly, before they are able to coalesce and take on permanency, and to the numerous domestic animals, primarily donkeys and dogs, which roam Makhanda’s streets, tearing open trash bags, and scattering litter. Failure to promptly clear illegal dumpsites has, as we described earlier, been linked to Makana’s own struggles, including a court order to remove them in 2021. Regardless, dumping remains a serious and growing problem in western Makhanda, primarily on marginal land, near access roads and near watercourses (Fig. 4). Although most dumpsites are self-contained,^33^ waste, primarily plastics, often blow around and settle in the natural environment, while existing dumpsites serve as magnets for additional dumping. Furthermore, although these residents are often able to adapt to a missed collection day, generally by bringing their bags back in from the kerbside and storing them until next week, bags that are left out, or not brought back in quickly enough, may be torn apart by one of the domesticated donkeys that roam the streets (a characteristic of Makhanda), or by dogs (in town), or cattle (on the outskirts). One Sunnyside resident^34^ explained this struggle, “the bloody donkeys and cows get to it before the van comes around, so you’ve got rubbish strewn all over and you need to collect and pick up”. The historic centre of town receives more attention from Makana, in terms of collection and street cleaning; however, residents decried what they saw as a proliferation of rubbish on the streets and in the gutters. Other busy areas within town have become popular dumping sites, such as near Shoprite,^35^ because sites are more regularly cleared out by municipal workers: i.e., residents who did not get collection often dump their waste there because they know it will be collected, as one resident^36^ confessed to doing, “we go throw our trash away by the containers at Shoprite when it has accumulated in the yard”.

**Fig. 5:**
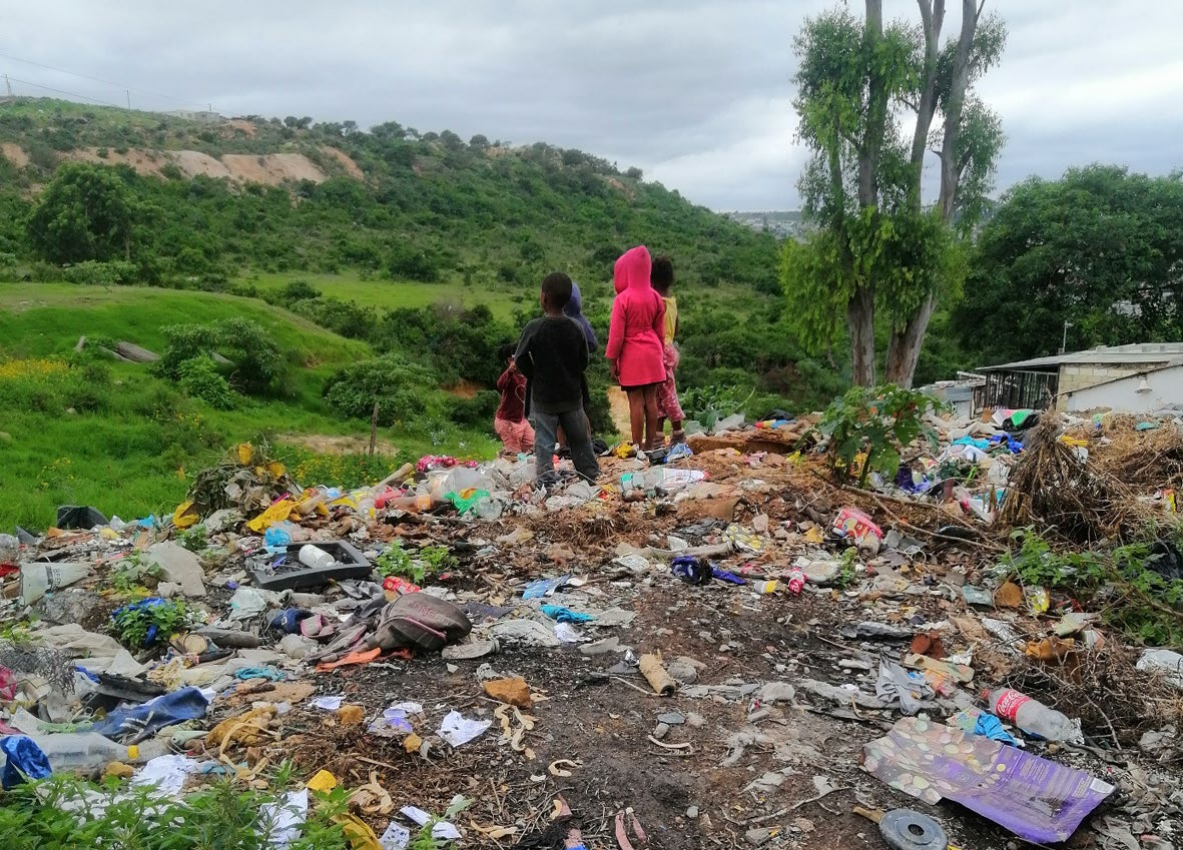
Joza children (Authors)

Joza has witnessed the same impacts, but to a much greater degree, with so much trash accumulating within public space and the natural environment that the community has, according to many respondents, come to resemble a dumpsite, or as one resident^37^ described, a “squatter camp”. What little public space that existed in the densely packed community has become choked with trash, with residents worried about the potential health impacts, as one respondent^38^ described, “the location is filthy, and when the location is dirty the environment is not okay. The air we breathe is not right….and we touch things we are not supposed to touch so it’s not okay for our health.” Respondents also decried a proliferation of pests in the community, including rats, which they attributed to the waste. However, the most acute concern was reserved for the community’s children who are forced to navigate polluted green spaces, and frequently play in and around dumping sites (Fig. 5), as one resident^39^ articulated, “it impacts us all health-wise. We have children that are playing in the street; they could get into medical waste, so it's a bit concerning.” Furthermore, because there is so much waste in the open environment, residents describe fighting a constant battle to keep their own homes and yards clear from a constant barrage of windborne plastics, paper, and other light-weight scraps. Despite the challenges, some residents described a growing apathy within Joza, with some community members no longer trying to maintain clean spaces, as one Joza woman^40^ described:

It has been a long-time, girl. It’s been a long time, and I won’t remember the year, but Grahamstown has long been suffering when it comes to rubbish. There are many dumping sites in the location, there is rubbish because the municipality does not collect, and there aren’t containers where people can go throw away their rubbish. So, community members don’t care anymore; they just throw things away wherever they can.

Although the day-to-day reality of living in a community that has come to resemble a dumpsite is harsh, many residents expressed a deeper fear that the situation may never improve, that the township as a dumpsite will become normalised, by the municipality and by those who live there, and residents will become so accustomed to living amongst trash, they will no longer see it:

The things I grew up thinking are weird, and now it is standard practice that you will be walking and there will be a diaper here and there. Children are now just jumping over rubbish because it's a daily occurrence. It has become normal, you know. The community no longer sees filth because we are living in filth.^41^

## Unclean and unequal

Although all Makhanda citizens have suffered from Makana’s retreat from regular public service provision, as we have shown, they have not suffered equally, with more affluent residents in the western neighbourhoods, largely able to ‘make a plan’ to get rid of their rubbish, and residents of the townships stuck living amongst their own waste, as it piles up higher and higher. In South Africa towns, where deep and persistent spatial inequalities linger, nearly thirty years after the start of democracy in 1994, Makhanda is a shining example of how the failure of South African municipalities to provide public services to historically disadvantaged communities, furthers the divide between the white and the non-white, the rich and the poor, complicating the state’s goal of upending intrenched inequalities, and creating cleaner, more equitable urban spaces.

In Makhanda, residents were keenly aware of this growing gulf. Although respondents from both of Makhanda’s halves expressed a sentiment that all residents were affected by Makana’s struggles, demonstrating some solidarity between communities, an unsubtle gulf remained between them, with western residents deploring the conditions of the townships, but distancing themselves from a space they have always seen, and prefer to keep, on the margins of their own community, and Joza residents looking west, to means of disposal, and to cleaner more affluent spaces that are not accessible or welcoming to themselves, as one resident of Joza^42^ neatly summed up:

In Grahamstown, the rich will always be able to dispose of their waste. They can either hire someone to come… maybe some people have got bigger yards where they can store it longer…. And when it comes to [Makana] prioritising, they start with the rich areas because the argument is that they are rate payers and our counter argument is that not everybody in the location is on Sbonelelo,^43^ we are also rate payers because we do not live in [government housing], we live in houses we have bought. You see? They do not prioritise us because apparently in the suburbs, rich people are more consistent in paying their rates, so they get prioritised. The municipality does not provide service for everyone [laughing].

Yet, in the same breath, while describing the inequality characterising Makhanda, the same resident^44^ reflected that although it was an unequal struggle, it was nonetheless shared amongst communities; a failing municipality may not affect all equally, but it does affect everyone:

Our municipality is one…. although you live in a better house… If you don’t have water in your house^45^ I also don’t have water in my house… Our problems are all the same, we drive on the same roads that have potholes. Whether you drive a posh car or a Corolla the roads are the same [laughing].

## Conclusion

**Fig. 6:**
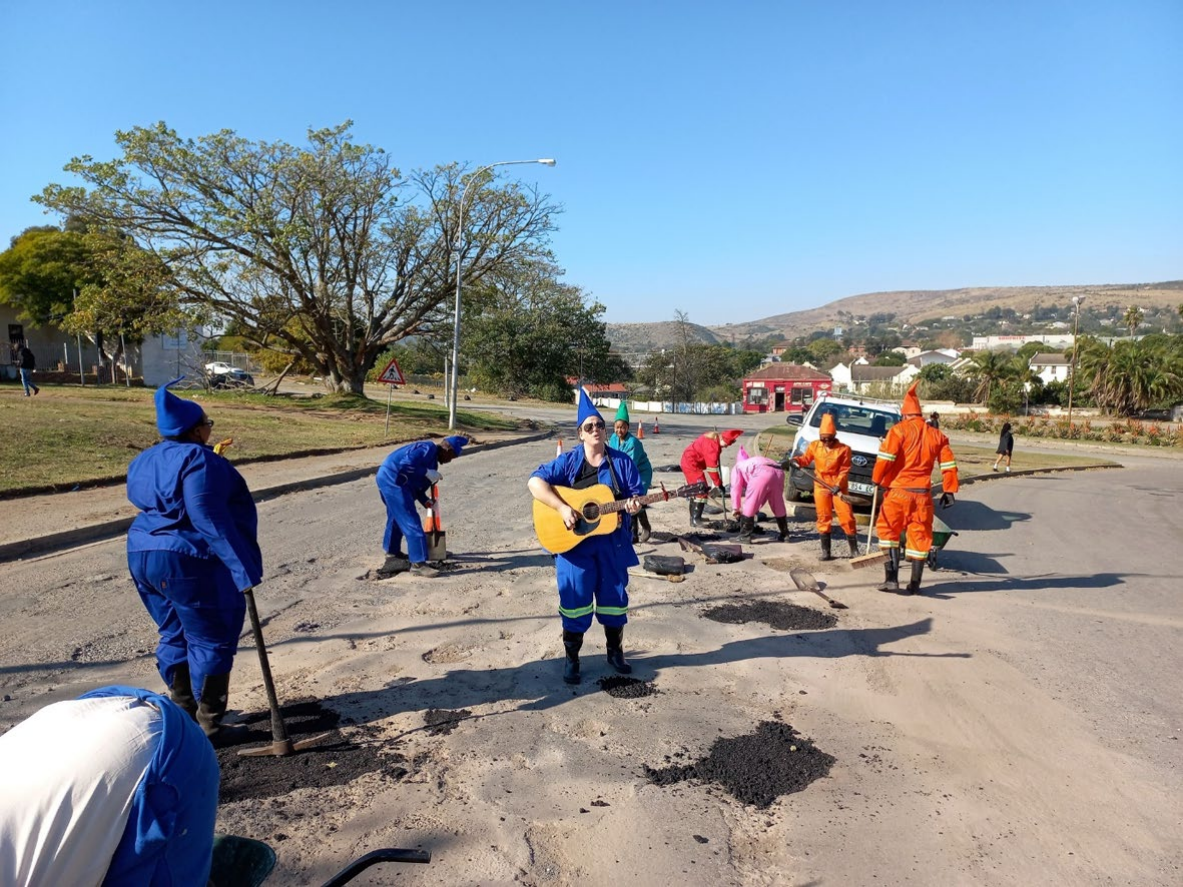
Performance art during the national arts festivals (Facebook: National Arts Festival Makhanda)

In the weeks leading up to the 2022 edition of Makhanda’s popular National Arts Festival, a strange site appeared on the city’s streets. Citizens, with pickaxes, shovels, and other tools, but dressed as brightly coloured gnomes and with an accompanying guitarist, worked to patch some of the city’s many potholes (Fig. 6). A few days later the gnomes appeared again, working to clear an illegal dumpsite: another in a series of community actions, leading up to the start of the festival. Organised by artist Gavin Krastin, the movement, of ‘queer artists-come-garden-gnomes’, in collaboration with local artisans and municipal workers, was a commentary on labour in a community in crisis. A series of actions centred around notions of ‘repair, community building, gratitude and transgressive joy’, in a city that could no longer fulfil its mandate, it was citizen service delivery as performance art.

South African municipalities hold the mandate for providing solid waste management services for millions of South Africans. However, a significant proportion of these municipalities appear to be on the brink of collapse. On the frontlines of municipal failure, the city of Makhanda, in Makana Local Municipality, following two decades of poor governance and mismanagement, has found itself unable to fulfil its mandate, with the state retreating on SWM service provision, and disruptions to waste management services becoming a daily reality. Set within this context of municipal failure, which remains uncharted territory in South Africa and abroad, this work explores how residents of Makhanda’s two halves: the affluent and predominantly white neighbourhoods in the west, and the poor, non-white townships in the east, have (or have not) adapted to manage and dispose of their own waste during periods of disruption. In an increasingly unequal and dysfunctional Makhanda, what does solid waste management look like?

The view from Joza is not reassuring. As the experiences of Makhanda’s residents have underscored, inequality underpins waste management systems, structuring who can or cannot access services, particularly in South Africa’s small towns where historic spatial inequalities remain unresolved. What this study has suggested is that while service delivery in Makana crumbles, and the state further retreats from its mandate to provide equal services to all communities, disadvantaged communities will be increasingly burdened with the detritus of their own existences; meanwhile more affluent residents are inconvenienced, but will manage to adapt to poorer services. Performance art may highlight the problem and show that citizens are willing to come together to address gaps left by the state, but it will not fill them equally, or resolve historic, entrenched inequalities. What viable alternatives to traditional centralised systems exist which do not perpetuate inequalities? Neoliberal alternatives to the state, such as privatised collection, processing, and disposal, are the most likely outlook. But, as Makhanda’s experience shows, and the literature has demonstrated (Mathekganye et al., 2019; Yates & Harris, 2018), such options are likely to contribute further to service delivery inequalities. Across South Africa, fostering waste markets while supporting informal waste workers, has the potential to increase the circularity of numerous recyclable fractions, and bolster the thousands of informal workers active within the country’s waste sector. In addition, funding decentralised, community-based solutions, which do not predicate on a robust state partner, may fill the emerging gaps, and exploring and articulating these pathways should be a key focus of future research and the national Waste RDI Roadmap, which is aimed at supporting South Africa’s transition to a circular economy (Schenck et al., 2022). Ultimately, however, a dramatic improvement in municipal governance is likely the smoothest pathway towards renewed, and hopefully, improved, service delivery. In South Africa, the likelihood of a sudden and significant improvement in municipal health seems low, although recent actions by the Auditor General have demonstrated that the national government is concerned about the nation’s municipalities and is willing to intervene when necessary. Yet, while the Covid-19 crisis, which exacerbated service delivery challenges across the country, seems to have passed its most critical point, the continued deterioration of the red-flagged municipalities since 2021 may suggest that the current national government lacks the means or the will^46^ to stem the tide of failure.

Lastly, it is important to emphasise that Makana is just one of many troubled municipalities on the tipping point in South Africa. The challenges experienced there reverberate across the nation, and the solutions scholars, practitioners and policymakers devise for them will hold broad national importance. While on a warming planet, and on a continent highly vulnerable to environmental change, the prospect of an evolving, more unpredictable climate, will put additional pressure on straining municipal systems, while making implementing innovative solutions even more challenging. Looking towards an uncertain future, the only thing we can be sure of is that there will be many more Makhandas. And without a clear plan for how to reverse the current tide of state failure, it is likely that few will be able to slow South Africa’s small towns from marching towards the precipice of a similarly tragic, unequal fate.

## Data Availability

The datasets generated during and/or analysed during the current study are available from the corresponding author on reasonable request.

## Footnotes

1 Residents also refer to the area as just the township or the location.

2 Metropolitan municipalities execute all the functions of local government for a city. South Africa has eight ‘Metros’, encompassing its largest cities, i.e., Johannesburg, Cape Town, Durban, etc.

3 According to the Auditor General (Mabuza, 2021), approximately R186 billion rand has disappeared from municipalities across the country over the past decade.

4 A report submitted by Cogta to the parliamentary local government portfolio committee in August 2021 indicated that a further 111 local municipalities were classified as being at medium risk for deteriorating further. Only 16 were rated as stable (Staff Writer, 2021).

5 See the February 21st, 2003, edition of Grocott’s Mail, the oldest surviving newspaper in South Africa, for an example of this type of reporting.

6 At least that we can identify.

7 A local newspaper. Founded in 1870, Grocott’s Mail is the oldest surviving newspaper in South Africa.

8 During this period Makana was administered by the province.

9 The lead opposition party within the municipality.

10 Specifically, Makana was ordered to “clear and eradicate seven specific illegal dumpsites within 14 days, work with communities to identify all other illegal dumpsites within 30 days, and then clear all illegal dumpsites within 120 days” (High Court of South Africa, 2021).

11 Town Interview #20, 16/08/2021.

12 Town Interview #18, 16/08/2021.

13 Joza Interview #5, 22/08/2021.

14 Town Interview #7, 08/08/2021.

15 Joza Interview #5, 22/08/2021.

16 Town Interview #1, 03/08/2021.

17 Town Interview #13, 12/08/2021.

18 Town Interview #15, 12/08/2021.

19 Joza Interview #3, 22/08/2021.

20 Town Interview #1, 03/08/2021.

21 A common South Africanism for a flat, grassy or bushy field.

22 Town Interview #12, 12/08/2021.

23 Within the few blocks of flats in Makhanda, residents dispose of their waste within shared containers within the complex. They are not responsible for final disposal, which is handled by whoever is managing the building. Respondents who lived in flats were superficially unaffected by disruptions, with the challenge of weekly disposal delegated to the management company.

24 Town Interview #16, 12/08/2021.

25 This is an assigned pseudonym.

26 Town Interview #1, 03/08/2021.

27 Town Interview #16, 12/08/2021.

28 Town Interview #14, 08/08/2021.

29 Town Interview #9, 08/08/2021.

30 Residents described paying a collection fee of R50 for five bags.

31 A handful of Joza respondents describing banding with neighbours to hire a truck for once-off trips to the dump when conditions become particularly unbearable.

32 Joza Interview #5, 22/08/2021.

33 Construction and demolition waste is one of the most common materials dumped.

34 Town Interview #7, 08/08/2021.

35 A large outlet of a national grocery store chain.

36 Town Interview #20, 16/08/2021.

37 Joza Interview #19, 05/09/2021.

38 Joza Interview #7, 22/08/2021.

39 Joza Interview #9, 25/08/2021.

40 Joza Interview #7, 22/08/2021.

41 Joza Interview #5, 22/08/2021.

42 Joza Interview #5, 22/08/2021.

43 An isiXhosa word referring to government assistance or benefits.

44 Joza Interview #5, 22/08/2021.

45 The resident is referring to a lack of water from widespread drought in the Eastern Cape (exaggerated by governance failings), not to localised gaps from poor/unequal provision.

46 And as the experiences of the Makana Citizens Front demonstrated in 2021 local elections, political change remains either a slow or unlikely outcome.
